# “Knowledge and attitudes of Spanish adolescent girls towards human papillomavirus infection: where to intervene to improve vaccination coverage”

**DOI:** 10.1186/1471-2458-14-490

**Published:** 2014-05-22

**Authors:** Pedro Navarro-Illana, Javier Diez-Domingo, Esther Navarro-Illana, José Tuells, Sara Alemán, Joan Puig-Barberá

**Affiliations:** 1 Facultad de Enfermería, Universidad Católica de Valencia “San Vicente Mártir”, C/Jesús, 10, 46007 Valencia, Spain; 2 Facultad de Medicina, Universidad Católica de Valencia “San Vicente Mártir”, C/Quevedo, 2, 46001 Valencia, Spain; 3 3FISABIO (Fundación para el Fomento de la Investigación Sanitaria y Biomédica), Av/Cataluña, 21, 46020 Valencia, Spain; 4 Cátedra Vacunología ‘Balmis’, UA–FISABIO, University of Alicante, Alicante, Spain

**Keywords:** Vaccines, Papillomavirus infections, Papillomavirus vaccines, Adolescent behavior, Health knowledge, Attitudes, Practice

## Abstract

**Background:**

HPV vaccine coverage is far from ideal in Valencia, Spain, and this could be partially related to the low knowledge about the disease and the vaccine, therefore we assessed these, as well as the attitude towards vaccination in adolescent girls, and tried to identify independently associated factors that could potentially be modified by an intervention in order to increase vaccine coverage.

**Methods:**

A cross sectional study was conducted in a random selection of schools of the Spanish region of Valencia. We asked mothers of 1278 girls, who should have been vaccinated in the 2011 campaign, for informed consent. Those that accepted their daughters’ participation, a questionnaire regarding the Knowledge of HPV infection and vaccine was passed to the girls in the school.

**Results:**

833 mothers (65.1%) accepted participation. All their daughters’ responded the questionnaire. Of those, 89.9% had heard about HPV and they associated it to cervical cancer. Only 14% related it to other problems like genital warts. The knowledge score of the girls who had heard about HPV was 6.1/10. Knowledge was unrelated to the number of contacts with the health system (Pediatrician or nurse), and positively correlated with the discussions with classmates about the vaccine. Adolescents Spanish in origin or with an older sister vaccinated, had higher punctuation. 67% of the girls thought that the vaccine prevented cancer, and 22.6% felt that although prevented cancer the vaccine had important safety problems. 6.4% of the girls rejected the vaccine for safety problems or for not considering themselves at risk of infection. 71.5% of the girls had received at least one vaccine dose. Vaccinated girls scored higher knowledge (p = 0.05).

**Conclusion:**

Knowledge about HPV infection and vaccine was fair in adolescents of Valencia, and is independent to the number of contacts with the health system, it is however correlated to the conversations about the vaccine with their peers and the vaccination status. An action to improve HPV knowledge through health providers might increase vaccine coverage in the adolescents.

## Background

Chronic infection with Human Papillomavirus (HPV) is known to cause a range of pre-cancerous, cancerous and being lesions like genital warts [1]. In Spain, cervical cancer is the sixth most common malignancy, representing 4.8% of all cancers in women. However its incidence is one of the lowest worldwide (Predicted age standardized rate in 2012 of 11.6 cases per 100.000 women/year) [2] and the mortality rate of 2.7 per 100.000 women/year, similar to European average [3,4].

HPV vaccines are highly efficacious in preventing chronic infections and dysplasias by serotypes 16 and 18, the most common oncogenic types [5-8].

Routine vaccination for all girls aged 14 years is recommended in Valencia, since October 2008, and available free of cost. However, the vaccine coverage decreased since its implementation. In 2008, 85%, of the target population received at least one HPV vaccine dose, and 73,9% received all three doses. The following year coverage dropped to 66.1% for one and 63.3% for three doses. Factors that could have influenced were: lack of awareness about the infection, the impact of the media in relation to some alleged safety problems at the beginning of the vaccination program [9], and concerns about vaccine effectiveness promulgated by local critical groups [10].

It is therefore important to develop activities in order to increase the vaccine coverage. As a first step we considered to assess the knowledge about the disease and vaccine and the attitudes towards the vaccine in the target groups, so that could devise interventions directed to the weaker points.

Worldwide many of the studies have examined individual awareness, knowledge and attitudes towards vaccination. Prior to the vaccine licensure, most of the studies reported as most significant a lack of knowledge, and recurrent myths, e.g. the impact of vaccination on promiscuity [11].

Subsequently, large vaccination campaigns in different countries have had a favorable impact on the perception of the vaccine to prevent disease. In the era post vaccine distribution, knowledge was positively associated with older age, higher education, number of sexual partners, previous diagnosis of cervical dysplasia or having ad an HIV test, and it was negatively associated in the United States with Latin American origin, opposition to premarital sex and certain religious beliefs [12,13].

In several studies, summarized in a systematic review there is a correlation between vaccine uptake and HPV disease and vaccine knowledge, attitudes regarding vaccination and having a healthcare provider as a source of information [14]. However [15] there is not strong evidence to recommend any specific educational intervention related to HPV vaccine for wide-spread implementation, as the quality of the trials published so far is low and in literate populations, therefore future studies are required to determine the effectiveness of culturally- competent interventions reaching diverse populations. In the same way, a metanalysis found that individual interventions (not during a health encounter) to inform or educate parents have a low impact on their babies vaccine coverage, although again the information is limited and of low quality, and the main recommendation was to do this communication as a complementary activity in a clinic [16].

There are few studies assessing the knowledge and attitudes of exclusively young adolescents towards vaccination. This age group is important as the primary target population for the vaccine delivery. At this age as girls become more independent, their decision to be or not vaccinated is, at least, as important as their parents’ [17]. At this age, less than 5% of the girls have had their first sexual intercourse [18], and therefore their sexual history, an important driver for higher knowledge, is less relevant.

A recent report from colleges in the United Kingdom, reported a low awareness and knowledge about HPV and HPV vaccination in a cohort of girls that were offered the vaccination through a school-based program [19].

The objective of our study was to assess the knowledge and attitudes of 15 year-old Spanish girls (who were candidates to receive the vaccine) towards HPV infection and the vaccine, and to identify independently associated factors that could potentially be modified by an intervention.

## Methods

### Design

A cross-sectional descriptive study in adolescent girls (aged 15, born in 1995) was carried out. All data was collected between September 2010 and May 2011. The girls were eligible to receive and complete the HPV vaccine series in the previous season (by June 2010).

Adolescent girls were recruited from primary schools of the Province of Valencia, Spain. Schools were randomly selected depending on the population for each of the Counties of the Province, and included private and public schools.

Both, mothers and girls completed an informed consent forms prior to any research activity. Investigators sent the informed consent to mothers through the school head teachers. The teachers collected the forms. Schools were paid €10 per each mother’s signed informed consent collected.

Adolescents completed the study questionnaire in the school, in the presence of one person from the research team. Prior to distribution all participants received the same information, and no further comments were made after the questionnaire was handed to the girls to avoid biasing the answers.

The Ethics Committee of the Center for Public Health Research (CSISP), Valencia, Spain approved the study.

### Instrument

A questionnaire, which consisted of 38 items, was developed. It assessed demographic information, social variables, including religious affiliation and religious practice, and social status of the family as recommended by the Spanish Epidemiology Association [20]. Use of health resources included the number of times in the previous year that they had a pediatric, GP, nurse or gynecologist consultation, and whether they were followed up due to any chronic condition. Toxic consumption: smoking, cannabis, cocaine and pills could have been ‘never consumed’, ‘consumed’, ‘seldom consumed’ or ‘habitual consumption’. Awareness of social problems was quantified using 5-points Likert scales (1 = no concern to 5 = extremely concerned) in 9 questions, including terrorism, epidemics, accidents, school failure and safety. Awareness of health problems was also quantified using the same Likert scales in 9 items, asking for concern about cancer, infectious diseases (i.e. AIDS and STDs), depression, new epidemics, diseases related to alcohol or other drugs and obesity. Two scores were developed, a social awareness score and a health awareness score. Each was calculated as the mean of the punctuation of the individual items (1 to 5).

HPV disease and HPV vaccine awareness were assessed by two items: ‘have you ever heard about HPV?’ and ‘have you ever heard about the HPV vaccine?’.

HPV knowledge was assessed using a 16 closed ended true/false items. By HPV knowledge we are referring to the degree to which a person possesses and understands objective information pertinent to HPV. To discourage guessing by the participants, researchers instructed them to choose ‘don’t know’. Distracter items, which assessed higher level of understanding of HPV infection, were included. These included an item to rule out the connection between HPV and ovarian cancer. We also included true/false statements (i.e. HPV produces hepatic cancer), to assess knowledge discrimination through rejection of false statements [21]. A knowledge summary score was computed by assigning one point for each correct response and zero points for each incorrect and ‘don’t know’ answers. Points were summed to create a knowledge summary score.

HPV vaccine knowledge was assessed with five questions: ‘How many doses are required for a complete vaccination schedule’ and four closed ended true/false items on the effect of the vaccine on future infections, the need to use condoms, the need to follow genital screening and the effect towards cervical cancer.

The means of receiving information about the vaccine was assessed with the question: ‘where have you heard about HPV vaccine?’.

Attitudes towards general vaccination were assessed by a Likert scale in two questions asking about their attitudes and their friends’, and also two open questions asking about their beliefs regarding benefits and problems of vaccines.

HPV vaccination attitude was assessed by asking the girls’ opinion about the vaccine, possible answers were: ‘it is a very good vaccine to prevent cervical cancer’, ‘it is a vaccine that prevents cervical cancer but has problems’, ‘it is a good vaccine but is painful and I am frightened’ and ‘it is a vaccine for a disease I will never get’.

We also asked about the sources for vaccine information (e.g., TV, press, radio, internet, brochures/leaflets, pediatrician, nurse, parents and friends). A general summary question asked the girls to consider and select the most relevant statement about the vaccine: ‘it is a very good vaccine to prevent cervical cancer’, ‘it is a vaccine to prevent cancer but it is problematic’, ‘it is a good vaccine but I am frightened because it hurts’, ‘it is a disease I am never getting and therefore I am not receiving the vaccine’. The answer to the number of vaccine doses received was checked with the Valencian vaccine registry, concordance was very high (Kappa = 0.92).

The questionnaire was piloted to validate comprehension among 15 girls in one school situated within a socio-economic deprived area and used to validate the comprehension of the questions.

### Statistical analysis

For qualitative variables, number and percentage with its normal 95% CI were calculated, with missing data included in the calculation of percentages. Non-parametric or ordinal variables were compared using the Mann–Whitney-Wilcoxon test when comparing two groups, or the Kruskal-Wallis test when comparing three or more groups. Correlations were estimated between the HPV knowledge summary score and all variables potentially related to knowledge. Pearson Product Moment Correlations were calculated to estimate the relationship between two continuous variables and Point-Biserial correlations were calculated to estimate the relationship between a dichotomous variable (e.g., provider recommendation for the HPV vaccine: yes or no) and a continuous variable (HPV knowledge).

All data processing was performed using SPSS for Windows (Version 15).

#### Questionnaire validation

Cronbach’s alpha was used to assess internal consistency of each construct of the questionnaire. When the alpha value was lower than 0.70, we considered that each of the individual questions from these constructs actually measured multiple unrelated psychological domains. Questions from these constructs were considered individually in the multivariate linear regression model of predictors of parental HPV vaccine acceptability.

## Results

### Sample

1278 mothers distributed among the schools were asked for informed consent, 833 (65.1%) accepted participation, in all those cases; their daughters fulfilled the questionnaire in the school and were included in the analysis.

Eighty percent of the adolescents lived with both parents and their socioeconomic status was similar to that occurring in Spain in 2010. Most of the parents (82.5%, 95% CI: 79.9-85.1%) were Spanish in origin and 11.3% from South America. Over 85% of the girls were born in Spain (Table 1).

**Table 1:** Characteristics of the study participants

	**N**	**%**
**Country of birth**		
Spain	716	86.0
Other	116	14
**Current family status**		
Living with both parents	658	79.0
Parents divorced/separated	138	16.5
Father or mother widow	25	3.0
Other	12	1.4
**Religious beliefs**		
Practising	117	0.14
Occasional practising	133	16.0
Non-practising	303	36.4
Non-believer	215	25.8
No answer	65	7.8
**Parents occupation**	**Father (%)**	**Mother (%)**
Unemployed	52 (6.9)	12 (1.6)
Undergraduate/director >10	187 (24.7)	117 (15.5)
Postgraduate/director <10	30 (4.0)	87 (11.5)
Admin./self-employed	171 (22.6)	100 (13.2)
Qualified technician	176 (23.5)	97 (12.8)
Semi-qualified technician	38 (5.0)	33 (4.4)
Unqualified worker	100 (13.2)	311 (41.1)

Study sample n = 833.

### Adolescents’ concerns and attitudes towards social and health problems

Among the social problems that girls were concerned about, school and sport accidents were the least rated, while those more severe or with higher media impact were of highest concern: road traffic accidents and terrorist attacks (Table 2). Concerns about health problems were diverse, being the weight problems the most rated (Table 2), with 78.5% (95% CI: 75.7-81.3%) of the girls being worried about it.

**Table 2:** Social and health concerns and toxic consumption among adolescent girls in Spain

	**%**	**95% CI**
Social-health worries*		
Road traffic accidents	82.8	80.2-85.4
Terrorist attacks	76.5	73.6-79.4
Weight problems	59	55.7-62.3
AIDS	78.5	75.7-81.3
STDs	73.7	70.7-76.7
**Toxic consumption**		
TOBACCO		
Never smoked	46.0	42.6-49.8
Consumed regularly	17.2	14.6-19.7
Alcoholic beverages		
At least once	82.2	79.6-84.8
Consumed regularly	31.5	28.3-34.6
Cannabis		
Never tried	79.4	76.6-82.2
Consumed regularly	3.9	2.6-5.2

*Percentage of responders that were worried or very worried with the social or health problems.

Tobacco consumption is high among the adolescent girls as 17.2% (95% CI: 14.6-19.7%) consumed it regularly (Table 2). Alcoholic beverages had been drunk, at least once, by 82.2% (95% CI: 79.6%-84.8%) and cannabis consumed regularly by 3.9% (95% CI: 2.6-5.2%). There was a high linear correlation between tobacco and cannabis consumption (p < 0.001). Tobacco consumption was unrelated to the concerns that girls had about cancer, alcohol or tobacco related diseases, weight problems, AIDS or STDs, as well as school failure or depression.

### Knowledge, attitudes and beliefs towards HPV infection and vaccination

The majority of the study participants (89.9%) had heard about HPV infection and knew its association to female genital cancer, and all of them associated HPV with cervical cancer; although 39.1% (95% CI: 35.8%-42.4%) also associated HPV with ovarian cancer. A lower percentage of girls had knowledge about the role of HPV infection among males with 5.5% (95% CI: 3.9%-7.0%) relating it with penis cancer and 26.9% (95% CI: 26.8%-33.0%) related it to a male infection (Table 3).

**Table 3:** Knowledge of adolescents in relation to HPV and HPV vaccine (n = 833)

	**Yes (%)**	**No (%)**	**Don’t know (%)**
**Diseases related to HPV**			
Penis cancer	46 (5.5)	628 (75.4)	159 (19.1)
Ovarian cancer	326 (39.1)	314 (37.7)	193 (23.3)
Genital warts	117 (14.0)	438 (52.6)	278 (33.4)
Cervical cancer	750 (90.0)	22 (2.6)	61 (7.3)
**Transmission**			
Lack of hygiene	29 (3.5)	683 (82.0)	121 (14.5)
Intimate kissing	19 (2.3)	706 (84.8)	108 (13.0)
Transmitted by boys	224 (26.9)	425 (51.0)	184 (22.1)
Sexual intercourse	718 (86.2)	26 (3.1)	89 (10.7)
**Ways of preventing HPV diseases**			
Hand washing	67 (8.0)	551 (66.1)	214 (25.7)
Good hygiene	429 (51.9)	273 (32.8)	130 (15.6)
Condoms	699 (83.9)	45 (5.4)	88 (10.6)
Cannot be prevented	104 (12.5)	389 (46.7)	338 (40.6)
**HPV vaccine**			
If vaccinated I will never get HPV infection	289 (34.7)	539 (64.7)	5 (0.6)
If vaccinated I will not need condom any longer in sexual intercourses	10 (1.2)	821 (98.6)	2 (0.2)
If vaccinated I will need cervical cancer screening	789 (94.7)	42 (5.0)	2 (0.2)
HPV vaccine completely protects against cervical cancer	211 (25.3)	612 (73.5)	10 (1.2)

Only 14% (95% CI: 11.6%-16.4%) of the girls knew the association between HPV and genital warts.

Most of the girls (86.2%; 95% CI: 83.8-88.5%) of the total sample were aware of the mechanism of transmission of the infection and 61% (95% CI: 57.7-64.3%) did not consider themselves to be at risk of infection.

With regards to potential ways of avoiding the diseases, 83.9% (95% CI: 81.4-86.4%) considered the use of condoms, 51.9% thought that an adequate personal hygiene avoided infection, and 12.5% (95% CI: 10.2-14.7%) considered that HPV infection could not be avoided.

The mean of the knowledge score, in those girls that had heard about HPV before the questionnaire, was 6.1 out of 10. It was positively related to the girl being Spanish in origin (p = 0.015) and having older sisters that had received the HPV vaccine (p = 0.016), and it was unrelated to their religious affiliation or health care attendance.

Most of the girls had heard about the HPV vaccine (97.4%; 95% CI: 96.3-98.5%), although some of them did not know what HPV was. With respect to beliefs and attitudes towards HPV vaccination, 539 (64.7%; 95% CI: 61.4-67.9%) knew that the vaccine potentially protected against any HPV infection and 25% (95% CI: 22.1-27.9%) thought that the vaccine completely protected against cervical cancer.

The utility of condoms, after vaccination, as protective barrier during sexual intercourse was reported by 821 (98.5%; 95% CI: 97.7-99.3%). Of the 833 girls, 789 (94.7%; 95% CI: 93.2-96.2%) thought that, despite vaccination, cervical screening was needed for secondary prevention.

### Do girls talk with their peers about the vaccine?

771 adolescents (92.6%; 95% CI: 90.8-94.4%) had recently spoken with classmates or friends about the vaccine. The most common topic was that the vaccine hurt (82.4%; 95% CI: 79.8-84.9%), it was very reactogenic (78.3%; 95% CI: 75.5-81.1%) and that it was a source of diseases (71.3%; 95% CI: 68.2-74.4%), but also that it was a very good vaccine to prevent cancers (73.1%; 9%CI: 70.1-76.1%). The overall positive comment among adolescents, that the HPV vaccine was very good, was a matter of discussion in 59.4% (95% CI: 56.1-62.7%) of the cases.

In general, adolescents concurred with the statement that the vaccine was very good for preventing cancers, 558 girls (67%; 95% CI: 63.8-70.2%); 22.6% (95% CI: 19.7-25.4%) felt that, although it prevented cancer, it had important problems. And 6.4% (95% CI: 4.7-8.1%) of the girls thought that they would definitely not have the vaccine because of the adverse events, and 3.2% (95% CI: 2.0-4.4%) because they were not at risk of infection.

There was a good positive correlation between adolescents discussing the vaccine with their peers and their knowledge score (p < 0.05).

### Health system utilization: impact on HPV knowledge and vaccine attitude

During the previous year, 699 girls (84%; 95% CI: 81.5-86.5%) had visited their GP or primary care pediatrician, 63% (95% CI: 59.7-66.3%) more than once; 51% (95% CI: 47.6-54.4%) attended a nurse clinic in primary care.

Of the 833 girls of the total sample, 12% (95% CI: 9.8-14.2%) were followed up for chronic conditions, primarily allergies and asthma and, less frequently, scoliosis or migraine.

A small percentage, 7% (95% CI: 5.3-8.7%), acknowledged not having received all the recommended vaccines during childhood or was unaware of their vaccination status.

In general, the adolescent population were positioned positively for vaccination, with only 1% (95% CI: 0.3-1.7%) completely against vaccination, 2.4% (95% CI: 1.4-3.4%) not being very positive, and 21.0% (95% CI: 18.2-23.8%) were indifferent towards vaccination with a very strong association between their belief on vaccination and their peers’ (p < 0.001).

The number of visits to the GP/pediatrician or nurse was unrelated to the knowledge score, the social and health awareness scores or their beliefs about HPV infection and the vaccine.

Vaccination status: 596 girls had received at least one vaccine dose at the time of the questionnaire (71,5%; 95% CI: 68.5-74,6%), and scored higher knowledge than non vaccinated (6.02 vs. 5.75; p = 0.05).

Only other two variables were significantly correlated with HPV knowledge: parents being of Spanish origin and having a sister that had been previously vaccinated. These variables were independently associated with the knowledge score and accounted for 5.8 of the variance in HPV knowledge score.

### Questionnaire validation

Internal reliability estimates were calculated for each construct and found to be adequate for the Social awareness, disease awareness and HPV knowledge with a Cronbach’s alpha between 0.86 and 0.78.

## Discussion

This study showed that adolescent girls in Spain had a fair knowledge about HPV infection and its prevention, with still important gaps, to remark the unbalanced perceived safety profile of the vaccine and that many do not consider themselves at risk of infection.

An important finding was the lack of relation between the girls’ knowledge and their attendance to the health system, and no correlation with the number of visits to either the pediatrician or the nurse, this meant that either the health provider did not invest time in discussing these facts or the explanation was not understood.

There are studies [22] where health care utilization is a positive predictor for vaccination, however there are areas where the provider recommendations are limited [23,24]. In Vadaparampil’s survey [24], over half of the pediatricians questioned in the United States had a low knowledge on HPV and perceived vaccine safety as a barrier for vaccination. However this finding was unrelated to their recommendation, as over 75% of the pediatricians followed the vaccine schedules, and recommended HPV vaccine in most of the visits for this age group of girls.

Unlike the impact of pediatricians or nurses on HPV knowledge, there is a strong correlation with peer discussions. This is an important finding as the information in this age group might come from less accurate sources and without quality filters. A review of Internet sources showed than both German and Spanish websites lacked balanced reporting on the completeness, transparency and exact dimensions of HPV and HPV vaccination [25]. Unbalanced media coverage may fuel vaccine alarm, and be shared among peers without any interaction with health care providers/system and may pose a risk to HPV vaccination programs.

In our study vaccinated girls scored higher in the knowledge construct, however whether higher knowledge was a driver for vaccination or the fact of being vaccinated suppossed higher knowledge (by instance the number of vaccine doses) is unknown. Adolescent and parents’ knowledge about HPV and HPV vaccination are frequently associated with vaccine uptake [14,26-28]. Although still not proven, an intervention to promote HPV vaccine knowledge may reduce one of the barriers for HPV vaccination. A recent German trial suggested that balanced information to the parents and girls, through brochures, increased the knowledge about HPV, but did not increase the vaccine uptake when compared to a group not receiving adequate information [29]. However, this last measurement, analyzed one year after the informative period, might be diluted by the mixing of both groups in the same classrooms, where information is shared and discussed. A systematic review that assessed the educational interventions used to increase HPV vaccine acceptance [15] showed that due to the quality of most of the trials and the population targeted, there is no evidence that increasing the knowledge about HPV does not improve vaccination coverage, therefore further studies of higher quality are required.

With the recommendation that vaccine be administered at age 14, young adolescent girls, may consider vaccination as one of their first health related decisions. In fact, although most of the times parents think that vaccinating their adolescent daughters should be their decision [30,31], in some studies parents agreed that a well-informed child should be able to request HPV vaccination without parental consent [17]. Therefore, timely knowledge about the disease and the vaccine seems to be an important factor for vaccine program success.

From our study, knowledge is unrelated to other health concerns like cancer or traffic road accidents, or to other social concerns like terrorism. It seems that the way adolescents look at HPV is rather unusual and it is possibly because HPV is new in their lives and, although they might have heard about the virus, there is little concern about it.

The only factors independently associated with knowledge were not modifiable, being Spanish in origin and having an older sister vaccinated. Spanish girls do not have an easier access to vaccines, as vaccines are given for free to all subjects living in Spain, therefore the explanation that immigrant population know less about the disease is not due to a lack of contact with the health system or lower education, as we passed the questionnaires in schools. There are descriptions in the USA of Latin American girls being less vaccinated, and it is possibly due to cultural or traditional barriers rather than having less facilities [32].

Our girls perceive HPV vaccination as a vaccine for STD more than a vaccine to prevent cervical cancer, and this is partially expected by their adolescent condition. Their attitude towards the vaccine is more intuitive than reasoned, in an exercise that mix correct and incorrect knowledges.

This study has some limitations. A systematic review [33] concluded that differences among studies results might be partly explained by variations in the questionnaires and lack of validation or analysis of consistency. We piloted the questionnaire to validate comprehension, however it is possible that not all girls fully understood all questions. We included questions previously validated in other studies and found that the internal consistency of the constructs was high.

Our response rate was not high, slightly over 65%, but the recruitment was difficult as we needed to obtain an informed consent from mothers and informed assent from the girls, and the former were difficult. The head teachers had to contact parents through the girls. We were unable to assess whether the responders differed from the non-responders, and that might have biased the results. School attendance is compulsory in Spain until the age of 16 therefore the universe of girls aimed for the questionnaire equaled the teenage population of Spain. In order to assess the representativity of our sample we compared our results with those of the National Institute of Statistics, and the distribution of our girls mirrored the general population in regards to the parent’s job [34] as well as the origin of the family. With reference of the toxic consumption we compared our results with the last published national health survey (2011–2012) [35].

In this survey data from women 15 to 24 years of age is aggregated, tobacco consumption in this group was 20,95%, slightly higher than our confidence interval. With regards to alcohol consumption, in the survey 20.5% of this group drank at least weekly, which is a lower figure that our sample’s answer to regular consumption. From the vaccination point of view, our sample had slightly higher vaccination coverage than the official figure for at least one vaccine dose (75% vs 66.5%). We considered that the sample was not very different to the general population as there was no a tendency to higher or lower toxic consumption, and also the age group we compared was not exactly the same. Therefore the fact that only 65% of the mothers signed the informed consent could bias the results but our estimation is that their representativity was high.

## Conclusion

In conclusion, knowledge about HPV and it prevention is fair in Spanish adolescents, although they had important gaps. Their information is unrelated to the number contacts with the health system but to their discussions with peers, which might pose the vaccination program at risk in case of spurious associations.

Acceptability might increase if misleading information is explained and discussed with health providers that might devote a longer time to explain the disease and vaccine to adolescents. However this activity should be further evaluated.

## Abbreviations

AIDS: Acquired immunodeficiency syndrome; CSISP: Center for public health research; GP: General practitioner; HIV: Human immunodeficiency virus; STD: Sexually transmitted diseases; USA: United States of America; HPV: Human papillomavirus.

## Competing interests

Díez-Domingo and Puig-Barberá were principal investigators in clinical trials sponsored by GlaxoSmithKlein, Merk and Sanofi Pasteur MSD. The rest of authors have no conflict of interests.

This study was sponsored by Sanofi Pasteur – MSD.

## Authors’ contributions

PN and JD-D made substantial contributions to conception and design, acquisition of data, analysis and interpretation of data and writting the manuscript. EN, JP, JT and SA were involved in drafting the manuscript or revising it critically for important intellectual content. All of them have approved the manuscript to be published.

## Authors’ information

At the moment, all of the authors are working in vaccines research projects and public health.

PN is the Dean of the Faculty of Nursing in the Catholic University of Valencia.

JD is the Research Director of the Vaccines Research Department in FISABIO, Valencian Government.

JT is the Director of the “Balmis” Chair of Vaccinology, in University of Alicante.

## Pre-publication history

The pre-publication history for this paper can be accessed here:

http://www.biomedcentral.com/1471-2458/14/490/prepub
